# Supplementary material for: Outliers (typically) cannot cause type I errors in one-sample/paired t-tests
Source: PLoS One. 2026 Feb 17;21(2):e0341720. doi: 10.1371/journal.pone.0341720 (PMC12912702; doi:10.1371/journal.pone.0341720)
Supplement: S1 Appendix A — (PDF) [file pone.0341720.s001.pdf]

Appendix A: Basic Derivations

New Sample Mean:

$$\begin{aligned}
\hat{\mu}' &= \frac{1}{n+1}\sum_{i=1}^{n+1} x_i = \frac{1}{n+1}\left[\sum_{i=1}^{n} x_i + (\hat{\mu}+\Delta)\right] = \frac{(n+1)\hat{\mu}+\Delta}{n+1} \\
&= \hat{\mu} + \frac{\Delta}{n+1}
\end{aligned}$$

New Sample Variance:

$$\begin{aligned}
n\hat{\sigma}^2_{x'} &= \sum_{i=1}^{n+1}(x_i-\hat{\mu}')^2 = \left(\sum_{i=1}^{n}(x_i-\hat{\mu}')^2\right) + (x_{n+1}-\hat{\mu}')^2 \\
&= \left(\sum_{i=1}^{n}\left((x_i-\hat{\mu}) - \frac{\Delta}{n+1}\right)^2\right) + \left(\hat{\mu}+\Delta-\hat{\mu}-\frac{\Delta}{n+1}\right)^2 \\
&= \sum_{i=1}^{n}(x_i-\hat{\mu})^2 - \frac{2\Delta}{n+1}\cancelto{0}{\sum_{i=1}^{n}(x_i-\hat{\mu})} + n\left(\frac{\Delta}{n+1}\right)^2 + \left(\frac{n\Delta}{n+1}\right)^2 \\
&= (n-1)\hat{\sigma}^2_x + \Delta^2\left(\frac{n}{(n+1)^2}+\frac{n^2}{(n+1)^2}\right) \\
&= (n-1) + \Delta^2\cdot\frac{n(n+1)}{(n+1)^2} = (n-1) + \frac{n\Delta^2}{n+1}.
\end{aligned}$$

Thus, the new sample variance is:

$$\hat{\sigma}^2_{x'} = \frac{(n-1)}{n} + \frac{\Delta^2}{n+1}.$$
