# Supplementary material for: Outliers (typically) cannot cause type I errors in one-sample/paired t-tests
Source: PLoS One. 2026 Feb 17;21(2):e0341720. doi: 10.1371/journal.pone.0341720 (PMC12912702; doi:10.1371/journal.pone.0341720)
Supplement: S2 Appendix B — (PDF) [file pone.0341720.s002.pdf]

Appendix B: Proof of Theorem 1

We start with the inequality:

$$t' \leq t.$$

Since both t and t' are positive this is equivalent to $t'^2 \leq t^2$ and substituting in derivations from Appendix 1 yields:

$$\frac{(n+1)\left(\hat{\mu} + \frac{\Delta}{n+1}\right)^2}{\frac{n-1}{n} + \frac{\Delta^2}{n+1}} \leq n\hat{\mu}^2$$

Multiplying by the denominator of the LHS and expanding the squared term yields

$$(n+1)\hat{\mu}^2 + 2\hat{\mu}\Delta + \frac{\Delta^2}{n+1} \leq n\hat{\mu}^2 \left(\frac{n-1}{n} + \frac{\Delta^2}{n+1}\right).$$

From here grouping terms by Δ gives the following quadratic expression.

$$\Delta^2(n\hat{\mu}^2 - 1) - 2(n+1)\hat{\mu}\Delta - 2(n+1)\hat{\mu}^2 \geq 0$$

Let:

$$a = n\hat{\mu}^2 - 1, \quad b = -2(n+1)\hat{\mu}, \quad c = -2(n+1)\hat{\mu}^2$$

Plugging these terms into the quadratic formula and simplifying yields:

$$\Delta^*_{lower} = \frac{(n+1)\hat{\mu} - \hat{\mu}\sqrt{(n+1)[(n+1) + 2(n\hat{\mu}^2 - 1)]}}{n\hat{\mu}^2 - 1}$$

and

$$\Delta^*_1 = \frac{(n+1)\hat{\mu} + \hat{\mu}\sqrt{(n+1)[(n+1) + 2(n\hat{\mu}^2 - 1)]}}{n\hat{\mu}^2 - 1}$$

From here, if we assume the denominator is positive ($\hat{\mu} > \frac{1}{\sqrt{n}}$) then the original inequality (and thus $t' \leq t$) holds whenever

$$\Delta^*_{lower} \leq \Delta \leq \Delta^*_1$$

Further, as Δ^*_{lower} is always negative under these assumptions, that condition is necessarily met (by the $\Delta \geq 0$ assumption), and we need only know that $\Delta \leq \Delta^*_1$
