# Supplementary material for: Outliers (typically) cannot cause type I errors in one-sample/paired t-tests
Source: PLoS One. 2026 Feb 17;21(2):e0341720. doi: 10.1371/journal.pone.0341720 (PMC12912702; doi:10.1371/journal.pone.0341720)
Supplement: S4 Appendix D — (DOCX) [file pone.0341720.s004.docx]

Appendix D: Non-normal Simulations

To verify that the data generating process does not affect the results of experiment 1, we ran two alternate experiments in which the generation process is modified. The first alternative uses a t-distribution ($df=1$) to generate a set of unscaled data ($\tilde{\mathbf{x}}$).As the mean and variance of the t-distribution cannot be controlled by the distribution parameters in the same way as the normal distribution (and in fact the variance is undefined for $df=1$) the generated is then scaled by

$$\mathbf{x=}\frac{\tilde{\mathbf{x}}\boldsymbol{-}\hat{\mu}_{\tilde{\mathbf{x}}}\boldsymbol{+}\mu}{\hat{\sigma}_{\tilde{\mathbf{x}}}}$$

where $\hat{\mu}_{\tilde{\mathbf{x}}}$ and $\hat{\sigma}_{\tilde{\mathbf{x}}}$ are the sample mean and sample standard deviation of $\tilde{\mathbf{x}}$ and $\mu$ is the desired mean which is randomly generated according to $U(0,1)$ as in experiment 1. From there, subsequent processes for evaluting $\mathbf{x}$ exactly mirror the steps in the original experiment and the analogous results are presented in Figure S1. The second alternate experiment uses the exact same processes of the first (including calculation of $\mathbf{x}$ from $\tilde{\mathbf{x}}$), but instead of generating the results from the t-distribution uses a deterministic sequence of extreme values (0,1). So each dataset is made up of 50% samples at the minimum and 50% samples at the maximum where the minimum and maximum are calculated to achieve the desired mean ($\mu$) and variance ($1$). The results of this experiment are displayed in Figure S2. Comparing Figures S1 & S2 with Figure 2 in the paper shows that the results of the three experiments are practically identical and do not depend on the data generating process. Close scrutiny also shows that where there are subtle differences between the results of the main experiment and either of these two, Figures S1 and S2 are exactly alike. This is because the original experiment generated the data according to $N(\mu,1)$, but did not scale the data to ensure that the sample mean equalled $\mu$ exactly.


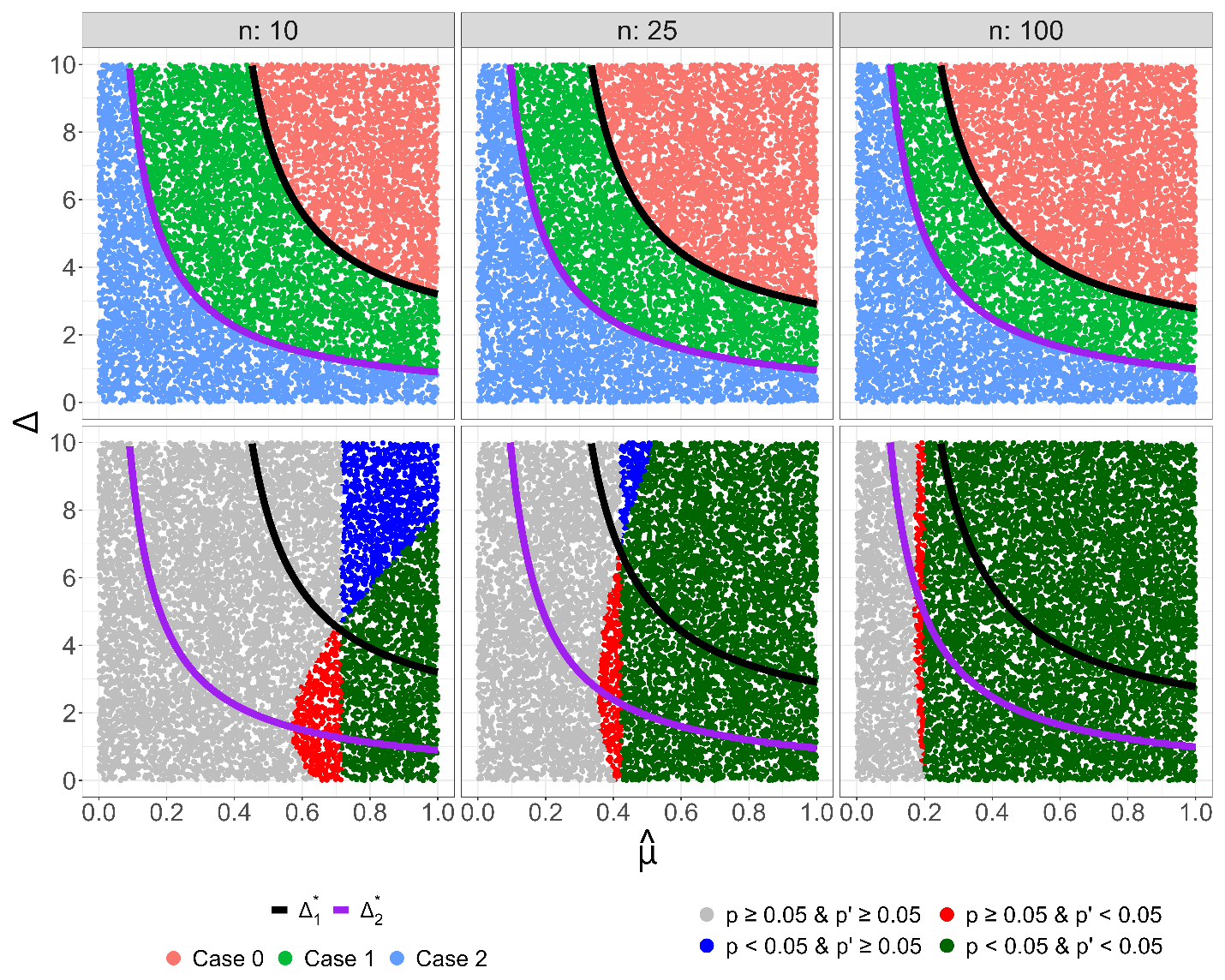


Figure S1: Results from experiment S1 which repeats experiment 1 using a data generated from a t-distribution with 1 degree of freedom.


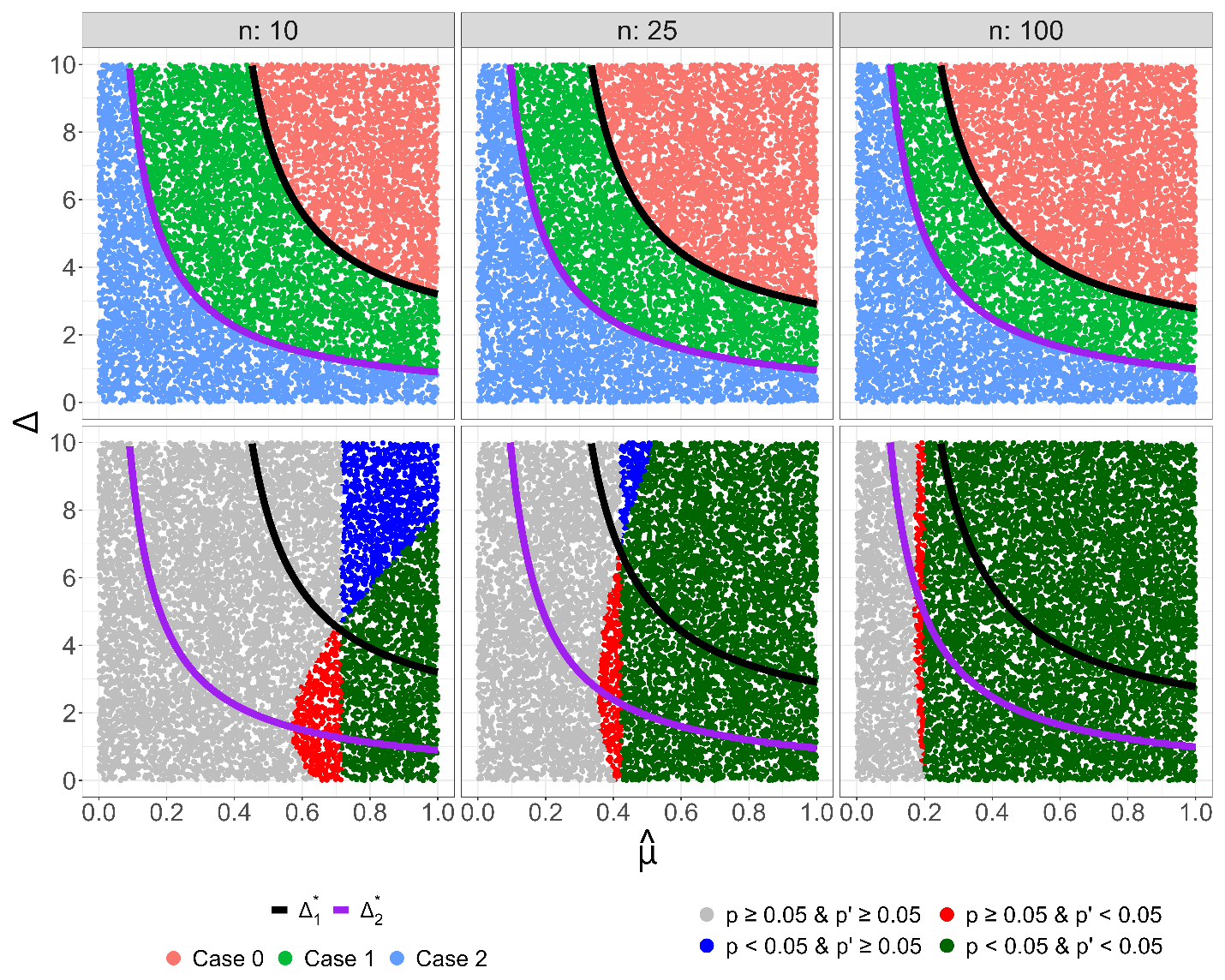


Figure S2: Results from experiment S2 which repeats experiment 1 using a data composed entirely of 0s and 1s rescaled to the desired mean / variance.
