# Supplementary material for: Outliers (typically) cannot cause type I errors in one-sample/paired t-tests
Source: PLoS One. 2026 Feb 17;21(2):e0341720. doi: 10.1371/journal.pone.0341720 (PMC12912702; doi:10.1371/journal.pone.0341720)
Supplement: Attachment — Submitted filename: t_plot.pdf [file pone.0341720.s005.pdf]

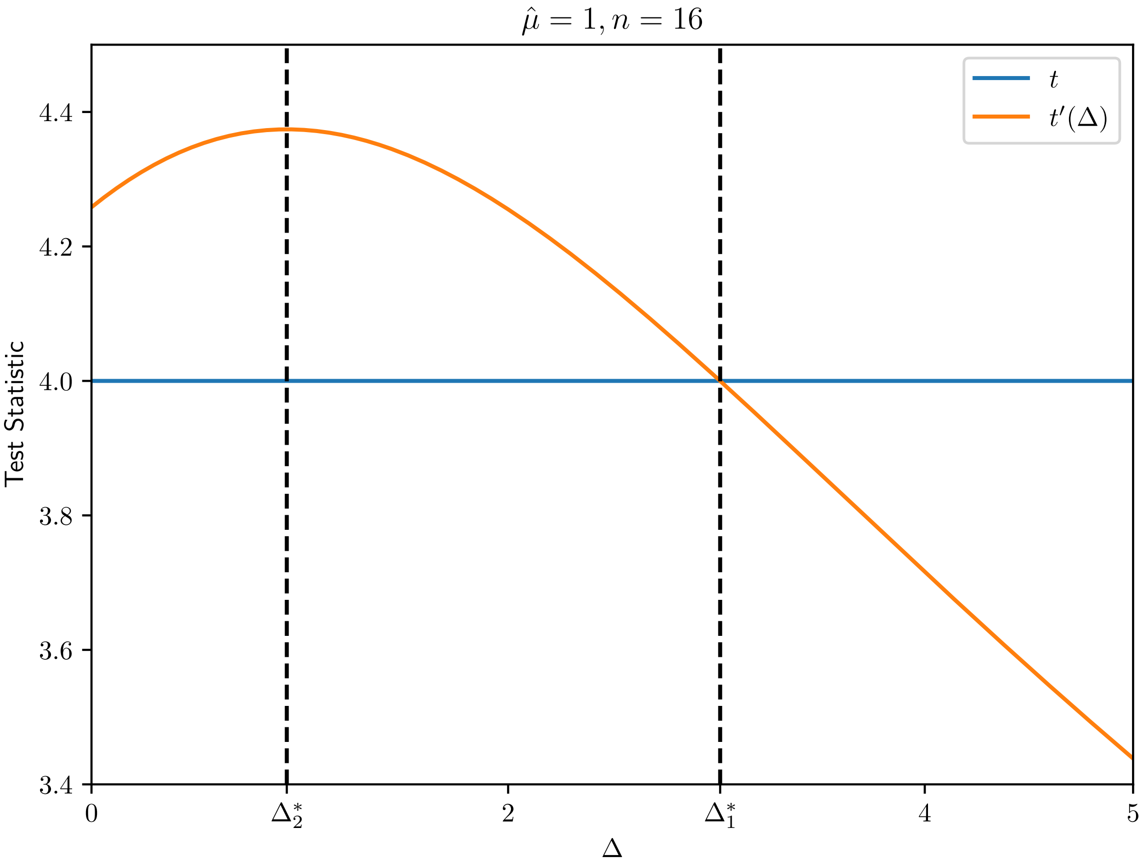

$\hat{\mu} = 1, n = 16$
t
$t'(\Delta)$
Test Statistic
4.4
4.2
4.0
3.8
3.6
3.4
0
Δ_2^*
2
Δ_1^*
4
5
Δ
